# Preoperative Education is Associated with Adherence to Downstream Components and Outcomes in a Colorectal Surgery Enhanced Recovery Program

**DOI:** 10.1097/AS9.0000000000000432

**Published:** 2024-05-16

**Authors:** Bayley A. Jones, Joshua Richman, Michael Rubyan, Lauren Wood, Alfonsus Adrian H. Harsono, Wendelyn Oslock, Nathan English, Burkely P. Smith, Robert Hollis, Larry R. Hearld, Isabel Scarinci, Daniel I. Chu

**Affiliations:** From the * Department of Surgery, University of Alabama at Birmingham, Birmingham, AL; † Department of Health Management and Policy, University of Michigan School of Public Health, Ann Arbor, MI; ‡ Department of Obstetrics and Gynecology, University of Alabama at Birmingham, Birmingham, AL; § Department of Quality, Birmingham Veterans Affairs Medical Center, Birmingham, AL; ‖ Department of Surgery, University of Cape Town, Cape Town, South Africa; ¶ Department of Health Services Administration, University of Alabama at Birmingham, Birmingham, AL.

**Keywords:** enhanced recovery adherence, enhanced recovery, ERP component adherence, preoperative education, outcomes

## Abstract

**Objective:**

This study evaluated the association between preoperative education and adherence to downstream components of enhanced recovery programs (ERPs) and surgical outcomes among patients undergoing elective colorectal surgery.

**Background:**

ERPs improve outcomes for surgical patients. While preoperative education is an essential component of ERPs, its relationship with other components is unclear.

**Methods:**

This was a retrospective cohort study of all ERP patients undergoing elective colorectal surgery from 2019 to 2022. Our institutional ERP database was linked with American College of Surgeons National Surgical Quality Improvement Program data and stratified by adherence to preoperative education. Primary outcomes included adherence to individual ERP components and secondary outcomes included high-level ERP adherence (>70% of components), length of stay (LOS), readmissions, and 30-day complications.

**Results:**

A total of 997 patients were included. The mean (SD) age was 56.5 (15.8) years, 686 (57.3%) were female, and 717 (71.9%) were white. On adjusted analysis, patients who received preoperative education (n = 877, 88%) had higher adherence rates for the following ERP components: no prolonged fasting (estimate = +19.6%; *P <* 0.001), preoperative blocks (+8.0%; *P =* 0.02), preoperative multimodal analgesia (+18.0%; *P <* 0.001), early regular diet (+15.9%; *P <* 0.001), and postoperative multimodal analgesia (+6.4%; *P <* 0.001). High-level ERP adherence was 13.4% higher (*P <* 0.01) and LOS was 2.0 days shorter (*P <* 0.001) for those who received preoperative education. Classification and regression tree analysis identified preoperative education as the first-level predictor for adherence to early regular diet, the second-level predictor for LOS, and the third-level predictor for ERP high-level adherence.

**Conclusion:**

Preoperative education is associated with adherence to ERP components and improved surgical outcomes.

## Abstract 2

Mini abstract

In this retrospective cohort study of 997 patients undergoing elective colorectal surgery in an enhanced recovery program (ERP), preoperative education was associated with improved process and outcome metrics. Preoperative education was associated with increased adherence to downstream ERP components, high-level ERP adherence (>70% of ERP components), and shorter length of stay.

## INTRODUCTION

Enhanced recovery programs (ERPs) are standardized perioperative care pathways that have been shown to decrease costs, length of stay (LOS), postoperative complications, and even reduce surgical disparities.^1–6^ The effectiveness of these programs relies on significant patient and care team engagement, as the benefits are modulated by adherence to the evidence-based components included in the programs.^7,8^ Studies suggest that ERP success in reducing postoperative symptoms, LOS, and readmission rates is driven by overall adherence to at least 70% of individual components.^7,9^ Despite the known impact of ERP adherence on outcomes, adherence rates to key ERP components are below 70% across hospitals in the United States, and implementation of ERPs with data capture protocols has resulted in minimal improvement in adherence.^10^ Therefore, it is important to identify contributors to adherence that may serve as modifiable targets to improve adherence rates and subsequently patient outcomes.

Various patient-level factors have been suggested to play a role in adherence, including socioeconomic status, race, and health literacy.^11,12^ However, it is unknown if patients receiving individual ERP components may impact the likelihood of them receiving subsequent downstream ERP components. Preoperative education is the first component of an ERP that a patient encounters and has been shown to increase patient preparedness, decrease patient anxiety, increase patient satisfaction, and improve patients’ overall surgical experience.^13–15^ ERP components, such as avoiding prolonged preoperative fasting and receiving a regional block, rely on patient understanding and engagement to be effective. Given this, patient education may play a critical role in adherence to downstream components as it empowers patients to play an active role in their care. However, the association of preoperative education with downstream ERP component adherence is unknown.

To address this knowledge gap, we examined the association of preoperative education with adherence to subsequent ERP components and surgical outcomes among colorectal surgery patients. We hypothesized that patients who receive preoperative education will have improved adherence to individual ERP components, especially ones that require the most patient engagement, as well as improved surgical outcomes.

## METHODS

### Study Design

**FIGURE 1.**
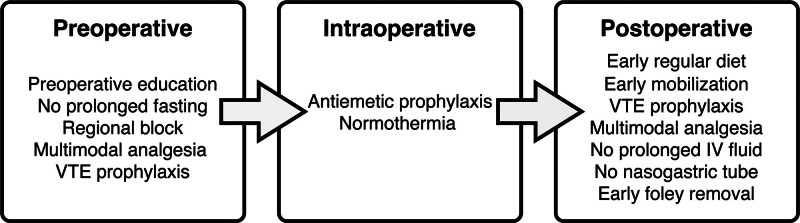
Individual ERP Components by Operative Phase. IV indicates intravenous; VTE, venous thromboembolism.

This study was a retrospective cohort analysis of colorectal surgery patients at the University of Alabama at Birmingham (UAB), a tertiary referral center serving a diverse rural and urban patient population. The study protocol was reviewed and approved by the UAB Institutional Review Board (IRB-300010279). ERP was implemented at UAB in December 2014 and is consistent with the 2018 Enhanced Recovery After Surgery (ERAS) Society Guidelines for elective colorectal surgery.^16^ Adherence to 14 individual ERP components is collected prospectively at the institution (Fig. 1), along with traditional demographic and clinical variables used by the American College of Surgeons National Surgical Quality Improvement Program (ACS-NSQIP). The study followed the Strengthening the Reporting of Observational Studies in Epidemiology (STROBE) reporting guideline.^17^

### Data Source and Study Population

The study cohort included all patients who underwent elective colorectal surgery at UAB from January 2019 to December 2022 and were included in our institution’s prospectively maintained ERP and ACS-NSQIP databases. All patients included in the study participated in our ERP, which included all 14 components in Figure 1. Exclusion criteria included patients under the age of 18 and patients undergoing urgent or emergent surgery. Only the first surgical encounter was included in the dataset for patients with more than one encounter during the study period.

### Exposure and Outcomes

The primary exposure of interest was self-reported receipt of preoperative education (one of the 14 ERP components included in the program). Verbal preoperative education is given in the surgery clinic by the surgeon, followed by a review of an ERP patient education booklet (written education) by the nursing staff. This booklet contains information regarding expectations and various components the patient may encounter, such as no prolonged fasting, early regular diet, and early ambulation. The measure is patient-reported at a subsequent preoperative anesthesia clinic visit during which patients are asked if they received verbal education, written education materials, or both. This information is recorded and extracted from the electronic medical record (EMR) by the ERP dashboard, and adherence is satisfied by receipt of either type of education. The primary outcome of interest was adherence to the 13 individual ERP components that occur after preoperative education (Fig. 1). Nonadherence may be due to provider preference or patient refusal. Secondary outcomes of interest included high-level ERP adherence (adherence to ≥70% of components), LOS, readmission within 30 days postoperatively, and the presence of any 30-day complication. ERP component adherence data was complete with the exception of the following missing data: early postop Foley removal (12.2%) and preop multimodal analgesia (1.7%).

### Statistical Methods

Unadjusted comparisons were made using 2-sided *t* tests for continuous or ordinal variables and χ^2^ tests for categorical variables. Analyses were repeated stratified by procedure type and unadjusted comparisons for procedure subgroups are included in Supplemental Tables 1–6, http://links.lww.com/AOSO/A348. Linear regression was used to model the association between preoperative education and adherence to each ERP component adjusted for other factors. Linear regression was used with dichotomous outcomes so that estimates could be interpreted as probabilities with additive effects. While unorthodox, this approach is valid with reasonably sized data sets and is more easily interpreted than the usual logistic regression model.^18^ All models were adjusted for race, gender, age, American Society of Anesthesiologists (ASA) class, procedure type, and indication in addition to any of the following that were significantly associated with the outcome at the *P <* 0.05 level: surgeon, hypertension, congestive heart failure, chronic obstructive pulmonary disease, smoking history, diabetes, dialysis, body mass index, operative approach, and steroid treatment.

The machine-learning method of classification trees, also called recursive partitioning, was used to examine associations between preoperative education and adherence to ERP components in a way that could identify important subgroups and potential interactions without prespecifying a model. For all trees, the models were set to build a complex tree that was then ‘pruned’ to a simpler tree that minimized the error rate from internal cross-validation. All analyses were done in R version 4.3.1.

## RESULTS

### Patient and Procedure Characteristics

A total of 997 patients met the inclusion criteria. Patient-level characteristics are detailed in **Table 1**. The mean (SD) age of the overall cohort was 56.5 years (15.8), 668 (57.3%) were female, and a majority were white (n = 717; 71.9%) with Black being the second most common race (n = 256; 25.7%). The most common indication for surgery was cancer (n = 356; 35.7%), followed by other benign (n = 309; 31.0%), diverticular disease (n = 169; 17.0%), and inflammatory bowel disease (n = 163; 16.4%). The majority of patients received a minimally invasive surgical approach (n = 669; 70.1%) *versus* open (n = 283; 28.4%). The majority of patients underwent a colectomy: 416 (41.7%) partial colectomies and 416 (41.7%) extended colectomies. Other patients received the following procedures: abdominoperineal resection/exenteration (7.6%), diverting loop ileostomy reversal (4.5%), colostomy reversal (1.2%), stoma construction (0.3%), small bowel procedures (0.9%), and other procedures (2.0%).

**TABLE 1.** 2019–2022 Colorectal Patient and Procedure Characteristics: Overall and by Preoperative Education

	Total (N = 997)	Preoperative Education (N = 877)	No Preoperative Education (N = 120)	*P* value
Age, mean (SD)	56.6 (15.7)	56.9 (15.2)	54.5 (18.6)	0.12
Sex, N (%)				0.40
Female	567 (56.9)	494 (55.7)	73 (60.8)	
Male	430 (43.1)	383 (43.7)	47 (39.2)	
Race, N (%)				0.49
White	717 (71.9)	638 (72.7)	79 (65.8)	
Black	256 (25.7)	218 (34.2)	38 (31.7)	
Unknown	16 (1.6)	14 (1.6)	2 (1.7)	
Asian	7 (0.7)	6 (0.7)	1 (0.8)	
American Indian/Alaska Native	1 (0.1)	1 (0.1)	0 (0.0)	
ASA class, N (%)				0.44
II	171 (17.2)	156 (17.8)	15 (12.5)	
III	785 (78.7)	685 (78.1)	100 (83.3)	
IV	40 (4.0)	35 (4.0)	5 (4.2)	
V	1 (0.1)	1 (0.1)	0 (0.0)	
Body mass index, mean (SD)	29.3 (7.6)	29.5 (7.5)	28.1 (8.4)	0.06
Comorbidities, N (%)				
Tobacco use	170 (17.1)	149 (17.0)	21 (17.5)	0.99
Steroid use	189 (19.0)	161 (18.4)	28 (23.3)	0.24
Hypertension	459 (46.0)	408 (46.5)	51 (42.5)	0.46
Diabetes				0.98
Insulin-dependent	49 (4.9)	43 (4.9)	6 (5.0)	
Non-insulin-dependent	114 (11.4)	101 (11.5)	13 (10.8)	
Congestive heart failure	25 (2.5)	20 (2.3)	5 (4.2)	0.34
COPD	35 (3.5)	33 (3.8)	2 (1.7)	0.29
ESRD	6 (0.6)	5 (0.6)	1 (0.8)	>0.99
Indication, N (%)				0.27
Cancer	356 (35.7)	320 (36.5)	36 (30.0)	
Other	309 (31.0)	265 (30.2)	44 (36.7)	
Diverticular	169 (17.0)	152 (17.3)	17 (14.2)	
Inflammatory bowel disease	163 (16.4)	140 (16.0)	23 (19.2)	
Procedure type, N (%)				0.30
Partial colectomy	416 (41.7)	353 (40.2)	63 (48.9)	
Extended colectomy	416 (41.7)	375 (42.9)	41 (45.1)	
APR/exenteration	76 (7.6)	70 (7.9)	6 (2.5)	
Stoma construction	3 (0.3)	3 (0.3)	0 (0.0)	
DLI reversal	45 (4.5)	41 (4.7)	4 (1.4)	
Colostomy reversal	12 (1.2)	10 (1.1)	2 (0.7)	
Small bowel	9 (0.9)	8 (0.9)	1 (0.4)	
Other	20 (2.0)	17 (1.9)	3 (1.0)	
Operative approach, N (%)				0.03
Minimally invasive	699 (70.1)	627 (71.5)	72 (60.0)	
Open	283 (28.4)	238 (27.1)	45 (37.5)	
Other	15 (1.5)	12 (1.4)	3 (2.5)	

APR indicates abdominoperineal resection; ASA, American Society of Anesthesiologists; COPD, chronic obstructive pulmonary disease; ESRD, end-stage renal disease; DLI, diverting loop ileostomy.

### ERP Adherence

Overall, 877 (88.0%) patients reported receiving preoperative education. Patients who reported receiving preoperative education were more likely to undergo a minimally invasive surgery (71.4% *vs* 60.0% for no preoperative education) but less likely to undergo open (27.1% *vs* 37.5%) or other procedures (1.4% *vs* 2.5%) (*P =* 0.03). Other patients and procedure-level characteristics were similar between patients who did and did not report receiving preoperative education (**Table 1**). Receipt of preoperative education did vary significantly by the surgeon (Supplemental Table 7, http://links.lww.com/AOSO/A348).

**Table 2** summarizes adherence to individual ERP components and outcome metrics. Adherence to the following components was higher among patients who reported receiving preoperative education *versus* those who did not: no prolonged fasting before surgery (44.4% *vs* 24.2%, respectively; *P <* 0.01), preoperative regional blocks (85.9% *vs* 75.8%; *P =* 0.01), preoperative multimodal analgesia (77.1% *vs* 60%; *P <* 0.01), early regular diet (77.3% *vs* 59.2%; *P <* 0.01), postoperative multimodal analgesia (97.7% *vs* 90.8%; *P <* 0.01), and early discontinuation of maintenance intravenous fluid (51.2% *vs* 39.2%; *P =* 0.02). Adherence to intraoperative normothermia was lower among patients who reported receiving preoperative education *versus* those who did not (9.2% *vs* 20.8%; *P <* 0.01). Of patients who reported receiving education, 62% had high-level ERP adherence *versus* 45.8% of patients who did not report receiving education (*P <* 0.01). Further, the mean LOS was 4.8 days among those who reported receiving education compared with 6.9 days for those who did not (*P <* 0.01). There were no statistically significant differences in complications or readmission rates between the 2 groups (Table 2).

**TABLE 2.** ERP Component Adherence and Outcomes For 2019–2022 Colorectal Surgery Patients: Overall and by Preoperative Education

	Total (N = 997)	Preoperative Education (N = 877)	No Preoperative Education (N = 120)	*P* value
ERP component				
No prolonged fasting	418 (41.9)	389 (44.4)	29 (24.2)	<0.01
Regional blocks	844 (84.7)	753 (85.9)	91 (75.8)	0.01
Preop multimodal analgesia	748 (76.6)	676 (77.1)	72 (60.0)	<0.01
Preop VTE chemoprophylaxis	530 (53.2)	476 (54.3)	54 (45.0)	0.07
Antiemetic prophylaxis	976 (97.9)	857 (97.7)	119 (99.2)	0.35
Intraoperative normothermia	106 (10.6)	81 (9.2)	25 (20.8)	<0.01
No nasogastric tube	862 (86.5)	762 (86.9)	100 (83.3)	0.36
Early mobilization	505 (50.7)	442 (50.4)	63 (52.5)	0.74
Early regular diet	749 (75.1)	678 (77.3)	71 (59.2)	<0.01
Postop multimodal analgesia	966 (96.9)	857 (97.7)	109 (90.8)	<0.01
Discontinuation of maintenance IVF	496 (49.8)	449 (51.2)	47 (39.2)	0.02
Postop VTE chemoprophylaxis	746 (74.8)	665 (75.8)	81 (67.5)	0.06
Early Foley removal	567 (56.9)	504 (57.5)	63 (52.5)	0.73
Outcome				
High-level ERP adherence	599 (60.1)	544 (62.0)	55 (45.8)	<0.01
LOS, Mean (SD)	5.1 (4.4)	4.8 (4.2)	6.9 (5.5)	<0.01
Readmissions	132 (13.2)	114 (13.0)	18 (15.0)	0.64
Complications	369 (37.0)	326 (37.2)	43 (35.8)	0.85

IVF indicates intravenous fluid; VTE, venous thromboembolism.

Adherence rates for multiple downstream components did vary significantly by surgeon, including no prolonged fasting, preoperative blocks, preoperative venous thromboembolism chemoprophylaxis, early regular diet, postoperative multimodal analgesia, and early Foley removal (Supplemental Table 7, http://links.lww.com/AOSO/A348).

### Adjusted Analysis

On adjusted analysis, patients who reported receiving preoperative education still had significantly higher adherence to several ERP components: no prolonged fasting (19.6% higher; *P <* 0.001), preoperative regional blocks (8.0%; *P =* 0.02), preoperative multimodal analgesia (18.0%; *P <* 0.001), early regular diet (15.9%; *P <* 0.001), and postoperative multimodal analgesia (6.4%; *P <* 0.001) (Table 3). In addition, high-level ERP adherence was 13.4% higher (*P <* 0.01) and LOS was 2.0 days shorter (*P <* 0.001) for patients who received education (Table 3).

**TABLE 3.** Linear Regression Models of ERP Component Adherence and Outcomes for Patients Who Received Preoperative Education Versus Those Who Did Not Receive Education Stratified by Procedure Type

	Overall cohort		Partial Colectomy		Extended Colectomy		Other	
Component	Coefficient	*P* value	Coefficient	*P* value	Coefficient	*P* value	Coefficient	*P* value
No prolonged fasting	0.196 (0.048)	<0.001	0.209 (0.067)	<0.01	0.182 (0.081)	0.02	0.033 (0.140)	0.81
Regional blocks	0.080 (0.035)	0.02	0.188 (0.049)	<0.001	−0.056 (0.055)	0.31	0.001 (0.106)	0.99
Preop multimodal	0.180 (0.041)	<0.001	0.310 (0.060)	<0.001	0.021 (0.067)	0.76	0.030 (0.121)	0.80
Preop VTE prophylaxis	0.069 (0.047)	0.14	0.085 (0.066)	0.20	0.080 (0.081)	0.32	−0.030 (0.122)	0.81
Normothermia	−0.113 (0.030)	<0.001	−0.164 (0.047)	<0.001	−0.065 (0.046)	0.16	−0.014 (0.072)	0.85
Early regular diet	0.159 (0.042)	<0.001	0.309 (0.059)	<0.001	0.002 (0.070)	0.98	0.060 (0.125)	0.63
Postop multimodal	0.064 (0.017)	<0.001	0.091 (0.027)	<0.001	0.053 (0.025)	0.04	−0.040 (0.042)	0.34
Discontinuation of IVF	0.094 (0.049)	0.05	0.109 (0.068)	0.11	0.026 (0.083)	0.76	0.016 (0.137)	0.91
Outcome								
LOS (days)	−1.99 (0.424)	<0.001	−2.21 (0.616)	<0.001	−1.16 (0.671)	0.08	-1.14 (1.221)	0.35
Readmission	-0.018 (0.033)	0.58	-0.044 (0.046)	0.34	0.013 (0.057)	0.82	0.054 (0.093)	0.56
Complication	0.002 (0.047)	0.96	-0.057 (0.064)	0.38	0.018 (0.079)	0.82	0.113 (0.125)	0.37
High-level adherence	0.134 (0.047)	<0.01	0.215 (0.066)	<0.01	-0.014 (0.080)	0.86	0.143 (0.133)	0.28

IVF indicates intravenous fluid; VTE, venous thromboembolism.

When adjusted models were stratified by procedure type (Table 3), partial colectomy patients who reported receiving preoperative education had higher adherence to the same ERP components as the overall cohort: no prolonged fasting (20.9% higher; *P <* 0.01), preoperative blocks (18.8%; *P <* 0.001), preoperative multimodal analgesia (31.0%; *P <* 0.001), early regular diet (30.9%; *P <* 0.001), and postoperative multimodal analgesia (9.1%; *P <* 0.001). In addition, high-level ERP adherence was 21.5% higher (*P <* 0.01) and LOS 2.2 days shorter (*P <* 0.001) for patients who reported receiving education. Extended colectomy patients who received preoperative education had higher adherence to no prolonged fasting (18.2% higher; *P =* 0.02) and postop multimodal analgesia (5.3%; *P =* 0.04). In contrast, the other procedure subgroup had no significant improvement in adherence or outcomes.

### Machine-Learning Classification Trees

**FIGURE 2.**
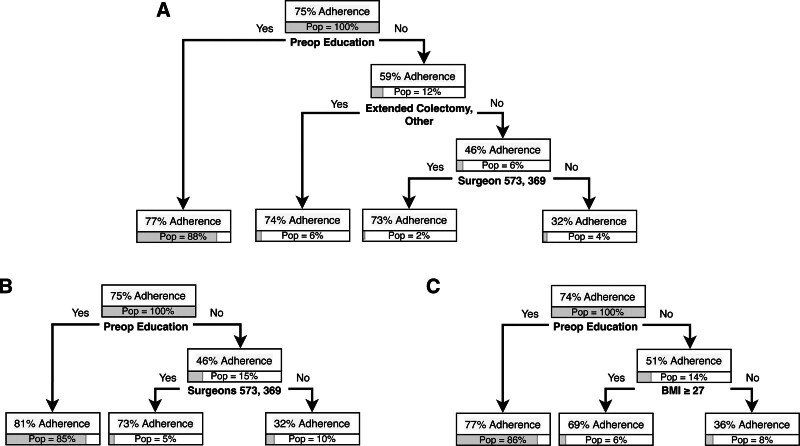
A, Early regular diet classification tree for overall cohort. B, Early regular diet classification tree for partial colectomy subgroup. C, Early regular diet classification tree for other subgroup. For example, (A) among patients who had preop education, adherence to early regular diet was 77%; this represents 85% of the overall cohort/subgroup. “Pop” indicates population and refers to the percentage of overall patients.

In parallel with traditional regression analyses, classification trees were used to identify factors associated with adherence to downstream components and outcomes. For the overall cohort, receipt of preoperative education was the first level and therefore most significant predictor in the early regular diet classification tree (Fig. 2A). Overall, 75% of patients were adherent to an early regular diet; this increased to 77% for those who reported receiving preoperative education (88% of population) and decreased to 59% for those who did not report receiving education (12% of population). Patients who did not receive education, and who underwent procedures other than extended colectomies or other procedures with 4 of the 6 surgeons had the lowest adherence at 32% (4% of population). Preoperative education was not a major predictor for other ERP components.

**FIGURE 3.**
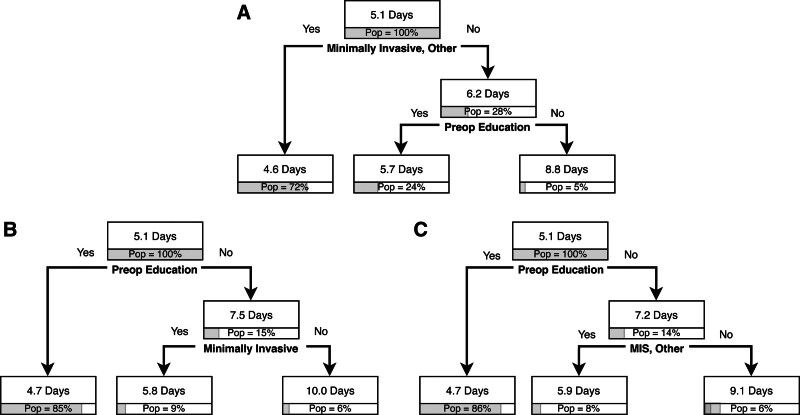
A, Length of stay classification tree for overall cohort. B, Length of stay classification tree for partial colectomy subgroup. C, Length of stay classification tree for other subgroup. For example, (A), among patients who had minimally invasive or other procedures, LOS was 4.6 days; this represents 72% of the overall cohort/subgroup. “Pop” indicates population and refers to the percentage of overall patients.

**FIGURE 4.**
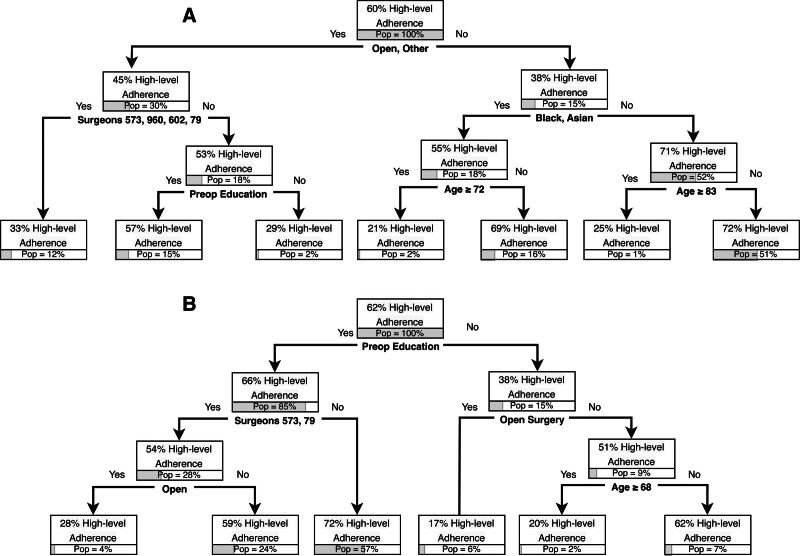
A, High-level ERP adherence classification tree for overall subgroup. B, High-level ERP adherence classification tree for partial colectomy subgroup. For example, (A) among patients who had open or other procedures, high-level adherence was 45%; this represents 30% of the overall cohort/subgroup. “Pop” indicates population and refers to the percent of overall patients.

Regarding outcomes, preoperative education was the second level of classification for LOS in the overall cohort (Fig. 3A), preceded only by the operative approach. For patients who underwent open procedures, the mean LOS was 6.2 days (28% of the population); however, if they received preoperative education (24% of population), LOS was 5.7 days *versus* 8.8 days if they did not receive education (5% of population). Preoperative education was the third-level predictor for high-level ERP adherence (Fig. 4A). Patients undergoing open or other procedures by 2 of the 6 surgeons and who received education had 57% high-level adherence (15% of population) *versus* 29% for those who did not receive education (2% of population). The readmissions and complications trees did not have preoperative education as a predictor.

**FIGURE 5.**
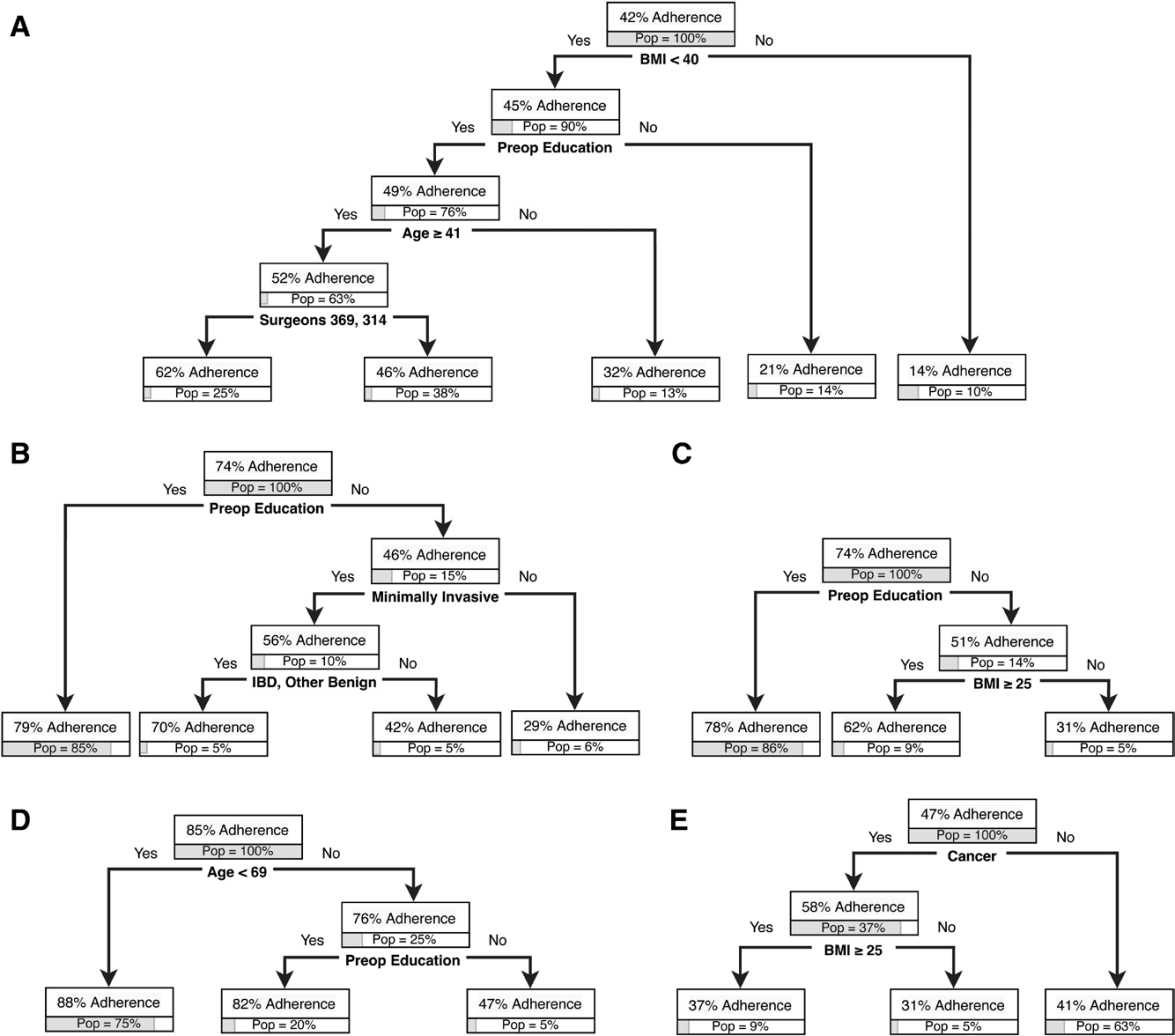
A, No prolonged fasting tree for partial colectomy subgroup. B, Preoperative multimodal analgesia for partial colectomy subgroup. C, Preoperative multimodal analgesia for other subgroup. D, No nasogastric tube placement for partial colectomy subgroup. E, Early discontinuation of maintenance intravenous fluids for partial colectomy subgroup. For example, (3A), among patients with BMI <40 and who had preop education, adherence to no prolonged fasting was 49%; this represents 76% of the overall cohort/subgroup. “Pop” indicates population and refers to the percent of overall patients.

On procedural subgroup analyses, preoperative education was found to be a high-order predictor for early regular diet for the partial colectomy (Fig. 2B) and other procedures subgroups (Fig. 2C); no prolonged fasting for the partial colectomy subgroup (Fig. 5A); preoperative multimodal analgesia for the partial colectomy (Fig. 5B) and other procedures subgroups (Fig. 5C); and no NGT placement (Fig. 5D) and early discontinuation of maintenance intravenous fluid (Fig. 5E) for the partial colectomy cohort. These procedural groups also had significant findings regarding postoperative outcomes. For partial colectomy patients, preoperative education was the first-level predictor in the LOS tree. LOS was 5.1 days for the overall cohort compared with 4.7 days if patients received education (85% of population) and 7.5 days if they did not receive education. The patients with the longest LOS were those who did not receive preoperative education and did not undergo a minimally invasive procedure (10 days, 6% of population) (Fig. 3B). Patients in the other procedures subgroup also had a shorter LOS if they received preoperative education at 4.7 days and 7.2 days if they did not receive preoperative education. Within the other procedure subgroup, patients who did not receive preoperative education and underwent open procedures had the longest LOS at 9.1 days (Fig. 3C). For high-level ERP adherence, preoperative education was the first-level predictor in the partial colectomy subgroup. High-level ERP adherence was highest for those who received preoperative education at 66% *versus* 38% for those who did not receive education. Patients who did not receive preoperative education and underwent open procedures had the lowest rate of high-level ERP adherence at 17% (Fig. 4B). Preoperative education was not a predictor in the complications or readmissions trees.

## DISCUSSION

Our study demonstrates that preoperative education is significantly associated with adherence to individual ERP components and outcomes. Patients who reported receiving preoperative education had higher rates of the following ERP components: no prolonged fasting, preoperative blocks, preoperative multimodal analgesia, early regular diet, and postoperative multimodal analgesia. In addition, high-level ERP adherence was higher and LOS was shorter for patients who reported receiving education. Machine-learning classification trees determined preoperative education to be an important factor associated with no prolonged fasting, preoperative multimodal analgesia, early regular diet, no NGT placement, and early discontinuation of maintenance intravenous fluid. With respect to outcomes, preoperative education was an important predictor of LOS in the overall, partial colectomy, and other cohorts, and high-level ERP adherence in the overall and partial colectomy cohort. Together, our analysis shows that preoperative education is an important predictor of increased adherence to downstream ERP components and is associated with improved surgical outcomes.

Preoperative education is recommended as an essential component of ERPs and has been shown to have many benefits for surgical patients.^16^ Patient-centered education is associated with decreased patient anxiety, decreased postoperative pain, and improved patient satisfaction.^19,20^ Studies have shown anxiety to be a common predictor of postoperative pain and postoperative analgesic consumption.^21–23^ Daltroy et al^24^ demonstrated the use of preoperative education in reducing the use of opioid pain medications, likely through a reduction in patient anxiety.^24^ Our study supports the argument that preoperative education can improve perioperative outcomes. To be specific, our study demonstrates that patients who reported receiving preoperative education had higher adherence rates to downstream ERP components, especially ones that require the most patient engagement. These findings build upon a prior study by Gillis et al^25^ in which patients identified preoperative education as a key component in enhancing patient engagement within ERPs. Patients reported that by understanding the ERP and the importance of the different components, such as multimodal analgesia and early regular diet, they could play an active role as knowledgeable partners throughout the surgical journey.^25^ In our study, we have demonstrated that preoperative education may serve as a powerful intervention to increase adherence to downstream ERP components through greater patient engagement.

The impact of preoperative education on outcomes extends beyond adherence to individual ERP components. Many studies have shown that high-level ERP adherence is critical to ERP success and improvement in surgical outcomes.^7^ Patients with high-level ERP adherence have a shorter LOS and lower risks of complications and postoperative symptoms.^1,7,8^ In this study, we show a novel association between preoperative education and high-level ERP adherence. Additionally, our study supports the previous finding of reduced LOS among ERP patients who receive preoperative education.^26^ To be specific, our study found that patients who reported receiving preoperative education had a reduction in LOS by almost 2 days. Furthermore, our machine-learning analysis independently identified preoperative education and operative approach to be 2 drivers of LOS. It is well-reported that patients undergoing minimally invasive surgery have significantly shorter LOS compared with open procedures.^27^ However, we demonstrated a novel finding of preoperative education as also being a predictor of LOS. It was the second-level predictor for the overall cohort after the operative approach and the first-level predictor for patients undergoing partial colectomies and other colorectal procedures. This suggests that education may mitigate some of the increases in LOS that we see in patients undergoing open procedures. Overall, we see that education may have a profound impact on surgical outcomes for ERP patients.

Our subgroup analysis demonstrated that preoperative education had the most impact on ERP component adherence and outcomes among the partial colectomy subgroup. One potential explanation for this is that the partial colectomy subgroup is the most uniform subgroup in our cohort. The variability in the extended colectomy and other subgroups may dampen the impact of preoperative education on outcomes. Another explanation may be that the education we are giving patients undergoing more involved procedures like total abdominal colectomies or colostomy reversals is not sufficient or comprehensive compared with the education we give to patients undergoing more straightforward partial colectomies. It has been demonstrated that patients undergoing partial colectomies experience more satisfaction and better functional outcomes than patients undergoing more extensive colectomies.^28^ If preoperative education for patients undergoing more extensive colectomies is not tailored to reflect the more complicated nature of the procedure with realistic postoperative expectations, education is less likely to make a significant impact on outcomes.

There are several limitations to this study. First, the generalizability of this study is limited as it is a single-institution study looking only at colorectal surgery patients. Additional studies are needed to determine the impact of education in other surgical specialties. Second, preoperative education is patient-reported and subject to recall bias and patient-level factors such as knowledge and health literacy. However, education is typically recorded in the EMR within a short timeframe (<1 month) from when education should occur, which should limit the impact of recall bias. In addition, we believe that it is more impactful to have education reported by the patient rather than the provider, as patient perception is critical in the context of education. Third, our preoperative education materials are not standardized. Patients receive similar written information regarding ERPs during their preoperative visit, but verbal communication can vary by provider.^29^ We did see differences in component adherence rates among surgeons. However, we controlled for this in the regression analysis and included surgeons in the classification trees to account for these differences. Fourth, our ERP practices continue to evolve, which could impact the results we see in this study. For example, early ambulation involves significant patient and nursing engagement, and we would expect education to play an important role in adherence. However, quality improvement efforts in ambulation are ongoing at our institution with subsequent improvements in adherence rates, which may contribute to the attenuated impact of education on ambulation. Fifth, the ERP data were extracted from the EMR, which leaves room for error with regard to adherence rates. However, the dashboard has been upgraded to ensure quality and accurate data extraction. In addition, the data were audited by a dedicated ERP team for any quality issues. Finally, our ERP dashboard extracts adherence data from the EMR for individual ERP components. However, we are unable to determine whether nonadherence is due to provider discretion or patient refusal. Additional qualitative studies will be needed to determine the main contributors to nonadherence to individual components.

The findings of our study have several implications. Although the majority of patients in our study received preoperative education, we should aim to have 100% of patients receiving education as this is an essential component of ERPs and quality surgical care. In addition, the findings of our study place significant emphasis on the need for future studies evaluating the various components of our current preoperative education practices. These components include who is involved in the education, when it is given, how it is delivered, and the content that is included. These studies would help to inform a more standardized approach to preoperative education and determine the most effective methods to increase patient understanding and engagement before surgery.

## CONCLUSIONS

In conclusion, our findings highlight the importance of preoperative education and its positive association with adherence to downstream ERP components and surgical outcomes. However, not all patients under ERPs receive preoperative education contributing to worse outcomes. Furthermore, the impact of preoperative education varies by procedure type. Opportunities exist to enhance the content and delivery of preoperative education to improve surgical care for all surgical populations.

## ACKNOWLEDGMENTS

The authors would like to acknowledge the UAB Enhanced Recovery Program Team and the UAB Enhanced Recovery Program Data Warehouse and Clinical Research Informatics teams.

B.A.J. contributed to the conception and design of the project, interpretation of data, and drafting and revision of the article. J.R. contributed to design of the project, analysis and interpretation of the data, and critical revision of the final manuscript. M.R. contributed to the conception and design of the project, interpretation of the data, and critical revision of the final article, B.A.J. L.W. contributed to design of the project, data acquisition, data analysis and critical revision of the final manuscript. A.A.H.H. contributed to design of the project, interpretation of the data, and critical revision of the final article. W.O., N.E., B.P.S., R.H., L.R.H., and I.S. contributed to interpretation of the data and critical revision of the final manuscript. No conflict of interest. D.I.C. contributed to the conception and design of the project, interpretation of data, and revision of the final article.

## Supplementary Material


