# Supplementary material for: Preoperative Education is Associated with Adherence to Downstream Components and Outcomes in a Colorectal Surgery Enhanced Recovery Program
Source: Ann Surg Open. 2024 May 16;5(2):e432. doi: 10.1097/AS9.0000000000000432 (PMC11191857; doi:10.1097/AS9.0000000000000432)
Supplement: Supplementary file 1 [file as9-5-e432-s001.pdf]

Supplemental Table 1. Partial Colectomy Subgroup: Patient and Procedure Characteristics Overall and By Preoperative Education

	**Total (N=416)**	**Preoperative Education (N=353)**	**No Preoperative Education (N=63)**	**P-value**
**Age,** Mean (SD)	56.4 (16.6)	56.5 (16.1)	55.6 (19.6)	0.69
**Sex,** N (%)				0.15
Female	233 (56.0)	192 (54.4)	41 (65.1)	
Male	183 (44.0)	161 (45.6)	22 (34.9)	
**Race,** N (%)				0.46
White	288 (69.2)	249 (70.5)	39 (61.9)	
Black	120 (28.9)	97 (27.5)	23 (36.5)	
Unknown	6 (1.4)	5 (1.4)	1 (1.6)	
Asian	2 (0.5)	2 (0.6)	0 (0.0)	
**ASA,** N (%)				0.05
II	70 (16.8)	66 (18.7)	4 (6.3)	
III	323 (77.6)	268 (75.9)	55 (87.3)	
IV	23 (5.5)	19 (5.4)	4 (6.3)	
**Body Mass Index**, Mean (SD)	30.1 (8.0)	30.4 (8.1)	28.0 (7.5)	0.02
**Comorbidities,** N (%)				
Tobacco Use	71 (17.1)	61 (17.3)	10 (15.9)	0.93
Steroid Use	104 (25.0)	84 (23.8)	20 (31.7)	0.24
Hypertension	209 (50.2)	181 (51.3)	28 (44.4)	0.39
Diabetes mellitus				0.97
Insulin dependent	25 (6.0)	21 (5.9)	4 (6.3)	
Non-insulin dependent	58 (13.9)	50 (14.2)	8 (12.7)	
Congestive Heart Failure	14 (3.4)	11 (3.1)	3 (4.8)	0.71
COPD	14 (3.4)	12 (3.4)	2 (3.2)	>0.99
Dialysis	2 (0.5)	2 (0.1)	0 (0.0)	>0.99
**Indication,** N (%)				0.84
Cancer	153 (36.8)	133 (37.7)	20 (31.7)	
Inflammatory Bowel Disease	89 (21.4)	74 (21.0)	15 (23.8)	
Diverticular	45 (10.8)	38 (10.8)	7 (11.1)	
Other	129 (31.0)	108 (30.6)	21 (33.3)	
**Operative Approach,** N (%)				0.03
Minimally invasive	307 (73.8)	268 (75.9)	39 (61.9)	
Open	109 (26.2)	85 (24.1)	24 (38.1)	

ASA indicates American Society of Anesthesiologists; DLI, diverting loop ileostomy; COPD, chronic obstructive pulmonary disease

Supplemental Table 2. Extended Colectomy Subgroup: Patient and Procedure Characteristics Overall and By Preoperative Education

	Total (N=416)	Preoperative Education (N=375)	No preoperative Education (N=41)	P-value
**Age,** Mean (SD)	57.3 (14.4)	57.6 (14.3)	54.8 (15.9)	0.24
**Sex,** N (%)				0.36
Female	246 (59.1)	225 (60.0)	21 (51.2)	
Male	170 (40.9)	150 (40.0)	20 (48.8)	
**Race,** N (%)				0.97
White	312 (75.0)	282 (75.2)	30 (73.2)	
Black	93 (22.4)	83 (22.1)	10 (24.4)	
Unknown	7 (1.7)	6 (1.6)	1 (2.4)	
Asian	3 (0.7)	3 (0.8)	0 (0.0)	
American Indian/Alaskan Native	1 (0.2)	1 (0.3)	0 (0.0)	
**ASA,** N (%)				0.53
II	75 (18.0)	67 (17.9)	8 (19.5)	
III	327 (78.6)	294 (78.4)	33 (80.5)	
IV	14 (3.4)	14 (3.7)	0 (0.0)	
**Body Mass Index**, Mean (SD)	29.2 (7.1)	29.5 (7.2)	26.8 (6.0)	0.02
**Comorbidities,** N (%)				
Tobacco Use	73 (17.6)	64 (0.3)	9 (22.0)	0.56
Steroid Use	60 (14.4)	54 (14.4)	6 (14.6)	>0.99
Hypertension	186 (44.7)	171 (45.6)	15 (36.6)	0.35
Diabetes mellitus				0.85
Insulin dependent	15 (3.6)	13 (3.5)	2 (4.9)	
Non-insulin dependent	40 (9.6)	37 (9.9)	3 (7.3)	
Congestive Heart Failure	6 (1.4)	5 (1.3)	1 (2.4)	>0.99
COPD	18 (4.3)	18 (4.8)	0 (0.0)	0.24
ESRD	1 (0.2)	1 (100)	0 (0.0)	>0.99
**Indication,** N (%)				0.18
Cancer	149 (35.8)	137 (36.5)	12 (29.3)	
Inflammatory Bowel Disease	37 (8.9)	34 (9.1)	3 (7.3)	
Diverticular	118 (28.4)	109 (29.1)	9 (22.0)	
Other	112 (26.9)	95 (25.1)	17 (41.5)	
**Operative Approach,** N (%)				0.32
Minimally invasive	326 (78.4)	297 (79.2)	29 (70.7)	
Open	89 (21.4)	77 (20.5)	12 (29.3)	
Other	1 (0.2)	1 (0.3)	0 (0.0)	

ASA indicates American Society of Anesthesiologists; DLI, diverting loop ileostomy; COPD, chronic obstructive pulmonary disease

Supplemental Table 3. Other Procedures Subgroup: Patient and Procedure Characteristics Overall and By Preoperative Education

	Total (N=165)	Preoperative Education (N=149)	No Preoperative Education (N=16)	P-value
**Age,** Mean (SD)	55.3 (16.1)	55.9 (15.4)	49.6 (21.2)	0.14
**Sex,** N (%)				0.31
Female	88 (53.3)	77 (51.7)	11 (68.8)	
Male	77 (46.7)	72 (48.3)	5 (31.3)	
**Race,** N (%)				0.23
White	117 (70.9)	107 (71.8)	10 (62.5)	
Black	43 (26.1)	38 (25.5)	5 (31.3)	
Unknown	3 (1.8)	3 (0.02)	0 (0.0)	
Asian	2 (1.2)	1 (0.01)	1 (6.3)	
**ASA,** N (%)				0.36
II	26 (15.8)	23 (15.4)	3 (18.8)	
III	135 (81.8)	123 (82.6)	12 (75.0)	
IV	3 (1.8)	2 (0.01)	1 (6.3)	
V	1 (0.6)	1 (0.01)	0 (0.0)	
**Body Mass Index**, Mean (SD)	27.6 (7.5)	27.1 (6.3)	32.1 (14.4)	0.01
**Comorbidities,** N (%)				
Tobacco Use	26 (15.8)	24 (16.1)	2 (12.5)	0.76
Steroid Use	25 (15.2)	23 (15.4)	2 (12.5)	>0.99
Hypertension	64 (38.8)	56 (37.6)	8 (50.0)	0.48
Diabetes mellitus				0.76
Insulin dependent	9 (5.5)	9 (0.1)	0 (0.0)	
Non-insulin dependent	16 (9.7)	14 (0.1)	2 (12.5)	
Congestive Heart Failure	5 (3.0)	4 (0.03)	1 (6.3)	0.42
COPD	3 (1.8)	3 (0.02)	0 (0.0)	>0.99
Dialysis	3 (1.8)	2 (0.01)	1 (6.3)	0.28
**Indication,** N (%)				0.77
Other benign	68 (41.2)	62 (41.6)	6 (37.5)	
Cancer	54 (32.7)	50 (33.6)	4 (25.0)	
IBD	37 (22.4)	32 (23.5)	5 (31.3)	
Diverticular	6 (3.6)	5 (0.03)	1 (6.3)	
**Operative Approach,** N (%)				0.19
Open	85 (51.5)	76 (51.0)	9 (56.3)	
Minimally Invasive	66 (40.0)	62 (41.6)	4 (25.0)	
Other	14 (8.5)	11 (7.4)	3 (18.8)	

ASA indicates American Society of Anesthesiologists; DLI, diverting loop ileostomy; COPD, chronic obstructive pulmonary disease

Supplemental Table 4. Partial Colectomy Subgroup: ERP Component Adherence and Outcomes

	**Total (N=416)**	**Preoperative Education (N=353)**	**No Preoperative Education (N=63)**	**P-value**
ERP Component				
No prolonged fasting	173 (41.6)	160 (45.3)	13 (20.6)	<0.01
Regional blocks	351 (84.4)	309 (87.5)	42 (66.7)	<0.01
Preop multimodal analgesia	302 (74.0)	273 (77.3)	29 (46.0)	<0.01
Preop VTE chemoprophylaxis	213 (43.2)	185 (52.4)	28 (20.1)	<0.01
Antiemetic prophylaxis	406 (97.6)	344 (97.5)	62 (98.4)	0.46
Intraoperative normothermia	58 (13.9)	41 (11.6)	17 (27.0)	<0.01
No nasogastric tube	354 (85.1)	306 (86.7)	48 (76.2)	0.05
Early mobilization	225 (54.1)	192 (54.4)	33 (52.4)	0.87
Early regular diet	314 (75.5)	285 (80.7)	29 (46.0)	<0.01
Postop multimodal analgesia	399 (95.9)	344 (97.5)	55 (87.3)	<0.01
Discontinuation of maintenance IVF	195 (46.9)	174 (49.3)	21 (33.3)	0.03
Postop VTE chemoprophylaxis	311 (74.8)	270 (76.5)	41 (65.1)	0.08
Early Foley removal	245 (68.6)	216 (61.2)	29 (46.0)	0.25
Outcome				
High-level ERP Adherence	257 (61.8)	233 (66.0)	24 (38.1)	<0.01
LOS, Mean (SD)	5.1 (4.6)	4.7 (4.2)	7.5 (5.8)	<0.01
Readmissions	53 (12.7)	41 (11.6)	12 (19.0)	0.15
Complications	139 (33.4)	116 (32.9)	23 (36.5)	0.67

VTE indicates venous thromboembolism; IVF, intravenous fluid

Supplemental Table 5. Extended Colectomy Subgroup: ERP Component Adherence and Outcomes

	Total (N=416)	Preoperative Education (N=375)	No Preoperative Education (N=41)	P-value
ERP Component				
No prolonged fasting	169 (40.6)	160 (94.7)	9 (5.3)	0.02
Regional blocks	362 (87.0)	325 (89.8)	37 (10.2)	0.63
Preop multimodal analgesia	326 (79.7)	294 (90.2)	32 (9.8)	0.94
Preop VTE chemoprophylaxis	206 (49.5)	189 (91.8)	17 (8.3)	0.36
Antiemetic prophylaxis	407 (97.8)	366 (89.9)	41 (10.1)	0.62
Intraoperative normothermia	35 (8.4)	29 (82.9)	6 (17.1)	0.22
No nasogastric tube	364 (87.5)	328 (90.1)	36 (9.9)	>0.99
Early mobilization	211 (50.7)	189 (89.6)	22 (10.4)	0.82
Early regular diet	320 (76.9)	289 (90.3)	31 (9.7)	0.99
Postop multimodal analgesia	406 (97.6)	368 (90.6)	38 (9.4)	0.07
Discontinuation of maintenance IVF	219 (52.6)	199 (90.9)	20 (9.1)	0.72
Postop VTE chemoprophylaxis	322 (77.4)	291 (90.4)	31 (9.6)	0.93
Early Foley removal	254 (66.5)	228 (89.8)	26 (10.2)	0.27
Outcome				
High-level ERP Adherence	262 (63.0)	236 (90.1)	26 (9.9)	>0.99
LOS, Mean (SD)	5.0 (4.2)	4.9 (4.0)	6.3 (5.1)	0.04
Readmissions	57 (13.7)	52 (91.2)	5 (8.8)	0.81
Complications	179 (43.0)	162 (90.5)	17 (9.5)	0.96

VTE indicates venous thromboembolism; IVF, intravenous fluid

Supplemental Table 6. Other Procedures Subgroup: ERP Component Adherence and Outcomes

	Total (N=165)	Preoperative Education (N=149)	No Preoperative Education (N=16)	P-value
ERP Component				
No prolonged fasting	76 (46.1)	69 (46.3)	7 (43.8)	>0.99
Regional blocks	131 (79.4)	119 (79.9)	12 (75.0)	0.76
Preop multimodal analgesia	120 (75.0)	109 (73.2)	11 (68.8)	>0.99
Preop VTE chemoprophylaxis	111 (67.3)	102 (68.5)	9 (56.3)	0.48
Antiemetic prophylaxis	163 (98.8)	147 (98.7)	16 (100)	0.62
Intraoperative normothermia	13 (7.9)	11 (7.4)	2 (12.5)	0.63
No nasogastric tube	144 (87.3)	128 (85.9)	16 (100)	0.14
Early mobilization	69 (41.8)	61 (40.9)	8 (50.0)	0.67
Early regular diet	115 (69.7)	104 (70.0)	11 (68.8)	>0.99
Postop multimodal analgesia	161 (97.6)	145 (97.3)	16 (100)	0.07
Discontinuation of maintenance IVF	82 (49.7)	76 (51.0)	6 (37.5)	0.45
Postop VTE chemoprophylaxis	113 (68.5)	104 (70.0)	9 (56.3)	0.41
Early Foley removal	68 (54.4)	60 (40.3)	8 (50.0)	0.22
Outcome				
High-level ERP Adherence	80 (48.5)	75 (50.3)	5 (31.3)	0.20
LOS, Mean (SD)	5.0 (4.5)	4.9 (4.4)	5.9 (5.3)	0.40
Readmissions	22 (13.3)	21 (14.1)	1 (6.3)	0.45
Complications	51 (30.9)	48 (32.2)	3 (18.8)	0.39

VTE indicates venous thromboembolism; IVF, intravenous fluid

Supplemental Table 7. Individual ERP Component Adherence by Surgeon

Component	Surgeon 573 (N=306)	Surgeon 960 (N=100)	Surgeon 369 (N=236)	Surgeon 79 (N=75)	Surgeon 770 (N=203)	Surgeon 314 (N=75)	P-value
Preop Education	283 (92.5)	81 (81.0)	213 (90.3)	62 (82.7)	174 (85.7)	63 (84.0)	<0.01
No prolonged fasting	135 (44.1)	43 (43.0)	105 (44.5)	22 (29.3)	74 (36.5)	39 (52.0)	0.04
Preop Blocks	276 (90.2)	80 (80.0)	194 (82.2)	64 (85.3)	162 (79.8)	66 (88.0)	0.01
Preop Multimodal Analgesia	232 (77.6)	72 (72.7)	180 (77.3)	48 (64.9)	153 (78.1)	61 (82.4)	0.14
Preop VTE Chemoprophylaxis	151 (49.4)	52 (52.0)	160 (67.8)	61 (81.3)	101 (49.8)	5 (6.7)	<0.001
Antiemetic Prophylaxis	301 (98.4)	96 (96.0)	233 (98.7)	74 (98.7)	197 (97.0)	73 (97.3)	0.58
Normothermia	22 (7.2)	10 (10.0)	27 (11.4)	5 (6.7)	30 (14.8)	12 (16.0)	0.05
No NG tube placed	253 (82.7)	87 (87.0)	213 (90.3)	61 (81.3)	182 (89.7)	64 (85.3)	0.07
Early Mobilization	158 (51.6)	52 (52.0)	121 (51.3)	25 (33.3)	105 (51.7)	42 (56.0)	0.07
Early Regular Diet	237 (77.5)	76 (76.0)	186 (78.8)	44 (58.7)	144 (70.9)	61 (81.3)	<0.01
Postop Multimodal Analgesia	303 (99.0)	91 (91.0)	230 (97.5)	71 (94.7)	195 (96.1)	74 (98.7)	<0.01
Discontinuation of Maintenance IVF	158 (51.6)	51 (51.0)	111 (47.0)	33 (44.0)	101 (49.8)	42 (56.0)	0.65
Postop VTE Chemoprophylaxis	230 (75.2)	82 (82.0)	182 (77.1)	52 (69.3)	150 (73.9)	50 (66.7)	0.19
Foley Removal	189 (69.2)	54 (63.5)	139 (67.8)	37 (56.9)	118 (69.4)	29 (45.3)	<0.01

VTE indicates venous thromboembolism; IVF, intravenous fluid
